# The probability density function of the surface electromyogram and its dependence on contraction force in the *vastus lateralis*

**DOI:** 10.1186/s12938-024-01285-1

**Published:** 2024-10-26

**Authors:** Javier Rodriguez-Falces, Armando Malanda, Cristina Mariscal, Silvia Recalde, Javier Navallas

**Affiliations:** 1 Department of Electrical and Electronical Engineering, Public University of Navarra D.I.E.E., Campus de Arrosadía S/N, 31006 Pamplona, Spain; 2 Department of Clinical Neurophysiology, Hospital Complex of Navarra, Pamplona, Spain

**Keywords:** Surface EMG, Filling factor, Probability density function (PDF), Gaussianity, Interference pattern analysis

## Abstract

**Introduction:**

The probability density function (PDF) of the surface electromyogram (sEMG) depends on contraction force. This dependence, however, has so far been investigated by having the subject generate force at a few fixed percentages of MVC. Here, we examined how the shape of the sEMG PDF changes with contraction force when this force was gradually increased from zero.

**Methods:**

Voluntary surface EMG signals were recorded from the *vastus lateralis* of healthy subjects as force was increased in a continuous manner vs. in a step-wise fashion. The sEMG filling process was examined by measuring the EMG filling factor, computed from the non-central moments of the rectified sEMG signal.

**Results:**

(1) In 84% of the subjects, as contraction force increased from 0 to 10% MVC, the sEMG PDF shape oscillated back and forth between the semi-degenerate and the Gaussian distribution.

(2) The PDF–force relation varied greatly among subjects for forces between 0 and ~ 10% MVC, but this variability was largely reduced for forces above 10% MVC.

(3) The pooled analysis showed that, as contraction force gradually increased, the sEMG PDF evolved rapidly from the semi-degenerate towards the Laplacian distribution from 0 to 5% MVC, and then more slowly from the Laplacian towards the Gaussian distribution for higher forces.

**Conclusions:**

The study demonstrated that the dependence of the sEMG PDF shape on contraction force can only be reliably assessed by gradually increasing force from zero, and not by performing a few constant-force contractions. The study also showed that the PDF–force relation differed greatly among individuals for contraction forces below 10% MVC, but this variability was largely reduced when force increased above 10% MVC.

## Introduction

The surface electromyogram (sEMG) signal is the algebraic summation of trains of motor unit potentials (MUPs) generated by active motor units. One possible strategy to analyze the sEMG signal is to calculate the probability density function (PDF) of the sEMG amplitudes. Indeed, the shape of the sEMG PDF has been utilized to separate contraction levels [1] or motions [2] as well as for prosthesis control [3] and to extract information of motor unit recruitment strategies [4].

There is not yet a general consensus on the shape of the sEMG PDF [5], nor is there an agreement on how this shape changes with contraction force [6]. The early studies on the sEMG PDF argued that a Gaussian density function can precisely model the sEMG PDF at various contraction strengths [7], and that this Gaussianity assumption holds even for very low contraction levels [8]. However, subsequent studies based on kurtosis analysis reported that, during low-intensity isometric contractions, the PDF is “more peaked near zero” and thus approximates more to a Laplacian distribution [9, 10]. On theoretical grounds, at low contraction forces (when the sEMG signal is not totally filled up), the sEMG PDF approximates to a Laplacian distribution, and then slowly shifts towards a Gaussian as force increases. Whereas this theory is supported by some previous studies [6, 11–13], there are yet some works indicating that the sEMG PDF does not always evolve towards Gaussianity as contraction force is increased [1, 14–16]. A recent study has proposed a Laplacian–Gaussian mixture model for surface EMG signals [5].

The above-mentioned studies suffered from 3 major methodological limitations in their attempt to characterize the sEMG PDF. First, in their protocols, force was not increased gradually and continuously from the zero level (as in a ramp contraction), but rather force was produced at a few fixed percentages of MVC (step-wise fashion) [11, 15, 16]. Second, the lowest force level examined in previous studies was 10% MVC [2, 6, 11]. Third, previous works only reported pooled results, and they did not investigate whether the dependence of sEMG PDF on contraction level varied greatly from one subject to another. Therefore, previous studies could not comprehensively examine how the sEMG PDF shape evolves with increasing contraction force, as they missed the information between the force levels, they overlooked the PDF data for forces below 10% MVC, and they disregarded the possible variability in the behavior of the PDF with contraction force among individuals.

**Fig. 1:**
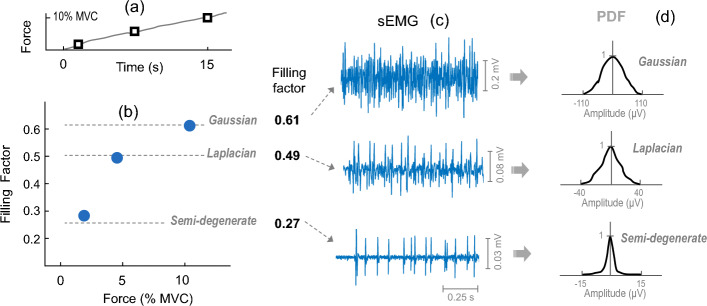
Representative example of how the filling factor (**b**), the sEMG signal (**c**), and the sEMG PDF (**d**) change as force was gradually increased (**a**) from the zero level in the *vastus lateralis*. **b** Filling factor values, calculated from short segments of the sEMG signal. **c** Three representative sEMG traces of 1-s duration recorded at different degrees of sEMG filling. **d** The PDF distributions corresponding to the selected sEMG signals

When force level is gradually increased from zero, the shape of the sEMG PDF changes progressively as more MUPs are incorporated into the sEMG signal (see Fig. 1 below and Fig. 4 in [17]). The change in the shape of the sEMG PDF can be investigated by a new analysis tool, called the filling factor, which is grounded on an analytical derivation of the EMG PDF [17]. In our previous studies, we showed that the filling factor tends to increase as the sEMG progressively fills up with MUPs, and the PDF shape evolves towards a Gaussian [17, 18]. To illustrate the parallel changes in the filling factor and the sEMG PDF as muscle activation increases, we show in Fig. 1 three sEMG signals selected at different degrees of sEMG filling in one subject. First, at very low contraction forces, when only a few MUP spikes are present in the sEMG signal, the filling factor takes a value close to 0.25, which corresponds to the semi-degenerate distribution. As force increases, additional MUPs come into play, and the filling factor increases to 0.5, the value corresponding to a Laplacian distribution. As force keeps increasing and the sEMG signal is close to filling up, the filling factor gradually approaches the value of 0.63, which represents Gaussianity. However, whether this behavior of the filling factor can be generalized to all individuals remains to be proved.

The shape of the sEMG PDF is influenced by anatomical factors (e.g., the subcutaneous layer thickness, spatial distribution of motor units throughout the muscle, and distribution of motor unit sizes), physiological factors (e.g., conduction velocity, fatigue), and neural factors (e.g., motor unit rate coding strategy) [15]. Because the above factors are subject-specific, one may expect to find a high variability in how the PDF shape changes with increasing force among subjects.

Several aspects underline the importance of testing the force levels below 10% MVC. First, examining low force levels is importance in prosthesis control where fine movements (i.e., hand gestures) requires exerting little force. Besides, many daily living activities involve muscle contractions below 10% MVC. Third, knowledge of the sEMG PDF at low force levels would be valuable to develop new algorithms to determine of the onset of myoelectric activity. Fourth, insight in the surface EMG signal and its PDF at low force levels would be of great interest in biofeedback experiments. From a physiological perspective, analysis of the filling factor at low forces will improve our understanding of motor unit recruitment patterns. Indeed, the filling factor analysis performed in our previous research allowed us to observe that, as force is gradually increased, a few large-amplitude MUP spikes, clearly standing out from the previous sEMG activity, may abruptly appear in the sEMG signal [18]. The abrupt appearance of such large-amplitude MUPs causes an abrupt marked change in the sEMG PDF shape. This observation may be indicative of the existence of large motor units with a very low recruitment threshold, thus partially challenging the orderly recruitment of motor units by size.

The objective of the present study was to elucidate how the shape of the sEMG PDF changes with contraction force by adopting three measures: (1) increasing force level gradually from zero, and not from 10% MVC; (2) assessing the variability in the relation of sEMG PDF to contraction force among subjects; (3) analyzing whether the force protocol utilized (force increased in a continuous manner vs in a step-wise fashion) has any impact on the PDF–force relation. It is hypothesized that a high variability exists in the PDF shape–force relation at low contraction forces, and that this variability would be reduced as force level increases.

## Results

### Representative examples of the changes in the sEMG PDF shape with contraction force

**Fig. 2:**
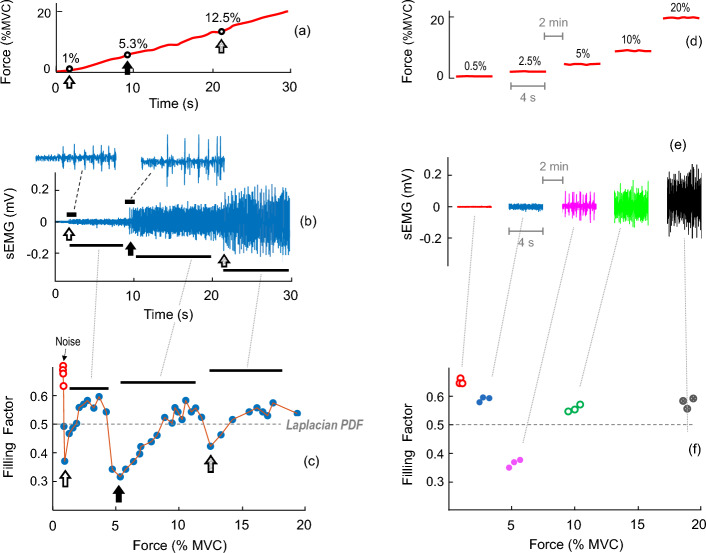
Impact of two different protocols of increasing contraction force on the sEMG signal and its PDF shape. In the example on the left panel, force was gradually increased from 0 to 20% MVC (**a**), whereas in the right panel force was increased in a step-wise fashion (**d**). The resulting sEMG signals are shown in plots **b** and **e**, and the corresponding filling factor values are shown in **c** and **f**. In plot **b**, the vertical arrows indicate the appearance of a few large-amplitude MUP spikes, clearly standing out from the previous sEMG activity. The onset of these prominent MUP spikes provoked an abrupt decrease in the filling factor (arrows in plot **c**). Note that the profile of the filling factor curve obtained when force was gradually increased (plot **c**) could not be accurately tracked when force was increased in constant-force steps (plot **f**)

Figure 2 shows two representative examples of sEMG signals recorded in one participant as force was increased in a continuous manner (left panel) and in a step-wise fashion (right panel). For each case, the force produced during the contraction is depicted at the top (plots a and d), and the filling factor values extracted from the sEMG signal are shown at the bottom (plots c and f). In the first protocol (left panel), as force was gradually increased, a few large-amplitude MUP spikes, clearly standing out from noise, abruptly appeared at ~ 2.0 s (see the white arrow and the inset in plot b). The onset of these new “large-amplitude” MUPs provoked an abrupt fall in the filling factor to ~ 0.35 (see the white arrow in plot c). As the participant kept on increasing force, prominent MUP spikes (clearly standing out from the previous sEMG activity) emerged again abruptly, first at ~ 9 s and then at ~ 21 s (see the black and grey arrows, respectively, in plot b), which caused abrupt decreases in the filling factor (see the black and grey arrows in plot c). Hence, the filling factor underwent several increases and decreases as force was gradually increased, and correspondingly, the PDF oscillated back and forth from the Gaussian to the semi-degenerate.

Differently, when force was increased in steps of constant force (right panel), the sEMG activity was rather “homogenous” within each force step (plot e), and thus the corresponding filling factor values calculated from each step were rather similar (plot f). Note that the filling factor values within each force level in plot f can be roughly considered as the result of sampling the “continuous” filling factor curve obtained when force was gradually increased (plot c). Because this sampling was “coarse” (only 5 force levels, 0.5, 2.5, 5, 10, and 20% MVC), the abrupt marked drops in the filling factor observed in plot c between 0.5 and 2.5% MVC (white arrow) and between 10 and 20% MVC (grey arrow) were missing (not detected) in plot f.

**Fig. 3:**
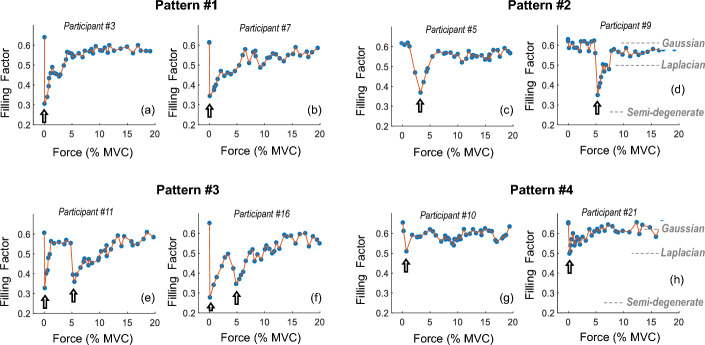
Illustrative examples of the changes in the filling factor as a function of contraction force in 8 different participants. Note the great diversity of behaviors of the filling factor with contraction force for forces below ~ 10% MVC. The vertical white arrows indicate the occurrence of an abrupt drop in the filling factor.

Figure 3 shows eight representative examples of the changes in the filling factor as a function of contraction force in different participants as force was gradually increased from zero level. It can be seen that the behavior of the filling factor with increasing force varied greatly among subjects for forces between 0 and ~ 10% MVC. Some illustrative patterns of variation of the filling factor are described next. In the first pattern, the filling factor decreased significantly and abruptly at the very onset of the contraction (at ~ 0.1% MVC, white arrows in plots a and b), and then increased progressively towards 0.63 (Gaussianity). In the second pattern, the abrupt decrease in the filling factor occurred at a higher force (3–6% of MVC force, white arrows in plots c and d). In the third pattern, two prominent abrupt drops in the filling factor were recognized (white arrows in plots e and f). In the fourth pattern, the filling factor decreased only moderately at the onset of the contraction (to ~ 0.50, white arrows in plots g and h), and then increased slowly towards 0.63.

**Fig. 4:**
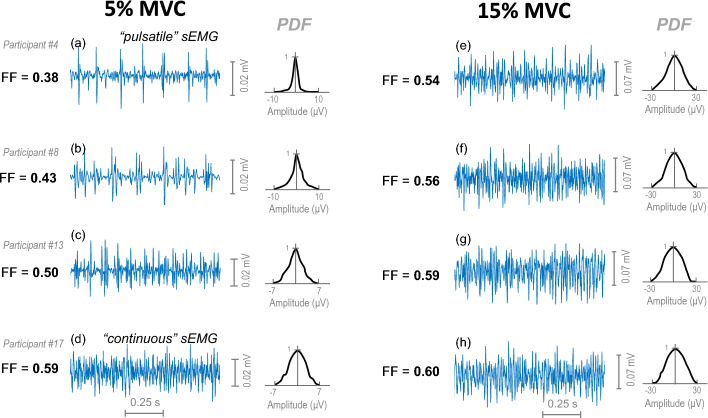
Representative examples of short segments of sEMG activity (1-s duration) recorded at 5% MVC (left panel) and 15% MVC (right panel) in different participants. The filling factor (FF) corresponding to each sEMG segment is indicated at the left of each trace, and the PDF is represented on the right. Note the high heterogeneity in the type of sEMG activity at 5% MVC. In contrast, all sEMG signals recorded at 15% MVC are rather similar, presenting a continuous “pattern”

Figure 4 shows representative examples of sEMG signals recorded at 5% MVC (first column) and at 15% MVC (second column) in four different participants. Note that the four signals recorded at 5% MVC showed different “types” of myoelectrical activity, ranging from a “pulsatile” activity (such as in plot a, where a few large-amplitude MUP spikes clearly stands out from the background sEMG activity; filling factor = 0.38) to a “continuous” activity (such as in plot d, where many MUPs greatly overlap with each other; filling factor = 0.59). By contrast, the 4 sEMG signals recorded at 15% MVC all presented a “continuous” activity, and the corresponding filling factor values were very similar (0.54, 0.56, 0.59, 0.60).

### Group analysis on the inter-subject variability in the relationship between filling factor and force

**Fig. 5:**
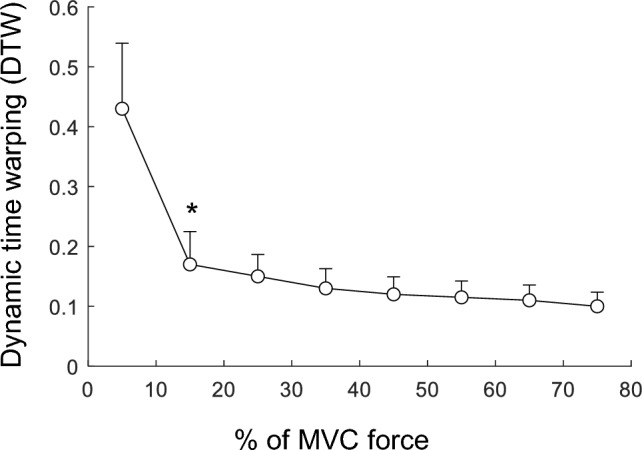
Average changes in the dynamic time warping (DTW) as a function of contraction force. Data are expressed as mean ± SD (N = 26). * Significantly different from the preceding force level at P < 0.05. Note that DTW was high (indicating high inter-subject variability in the filling factor–force relation) only for the (0–10% MVC) range

The great diversity of behaviors of the filling factor with contraction force among subjects only occurred for forces below ~ 10% MVC. This can be appreciated in Fig. 5, which shows the average DTW distances (obtained after comparing each subject’s curve and the average curve) corresponding to the force intervals (0–10), (10–20), (20–30), (30–40), (40–50), (50–60), (60–70), and (70–80)% MVC. It can be seen that DTW distance was significantly higher for the (0–10% MVC) force range than for the (10–20% MVC) range [*p* < 0.001, effect size Cohen’s *d* = 1.2 (large)]. Moreover, DTW distance was similar and very low for the rest of force intervals above 10% MVC [*p* > 0.05, effect size Cohen’s *d* < 0.1 (trivial)]. Collectively, these results indicated that the inter-subject variability in the relationship between filling factor and force was meaningful only for the (0–10% MVC) range.

In 84% of the subjects, we found marked fluctuations of the sEMG PDF shape between the semi-degenerate and Gaussian distributions. More specifically, 1 marked fluctuation occurred in 54% of subjects, whereas 2 marked fluctuations were observed in 30% of the cases.

### Group analysis on the effects of contraction force on the sEMG PDF

**Fig. 6:**
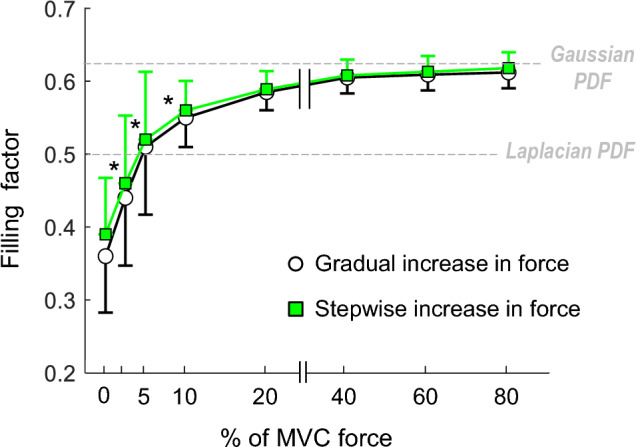
Average changes in the filling factor as a function of contraction force. Data are expressed as mean ± SD (N = 26). * Significantly different from the preceding force level at P < 0.05. Noteworthy, the filling factor had high variability for contraction forces below 10% MVC, but low variability for forces above 10% MVC

Figure 6 shows the average changes in the filling factor as a function of contraction force for the whole study group. It can be seen that the filling factor increased significantly and rapidly as contraction force increased from 0 to 10% MVC (Fig. 6, *p* < 0.001), and then this increase decelerated at higher force levels, slowing approaching the 0.63 level (Gaussian PDF). No significant force-protocol × contraction–force interaction was found (*p* = 0.37). Note the high variability in the filling factor for contraction forces below 10% MVC (long vertical bars), and the low variability for forces above 10% MVC (short vertical bars).

## Discussion

The main findings of the present study were the following:

1. When force was increased in a step-wise fashion, one can only sample the filling factor curve at specific values of force, thereby obtaining a limited restricted view of the changes in the filling factor curve.
2. In 84% of the subjects, the filling factor did not increase monotonically towards 0.63 (Gaussianity) as contraction force increased from 0 to 10% MVC, but rather it increased and decreased several times within such force range.
3. The behavior of the filling factor with contraction force varied greatly among subjects as force increased from 0 to 10% MVC, and this variability was largely reduced for forces above 10% MVC.
4. The pooled analysis of data indicated that the “average” filling factor increased rapidly for forces up to ~ 10% MVC, and this increase slowed down for higher forces, slowly approaching the 0.63 level (Gaussianity).

### Impact of the force protocol: force increased in a continuous manner vs in a step-wise fashion

We found that how contraction force was increased (continuously vs in a step-wise fashion) critically influences the information available in the resulting filling factor–force curve. Indeed, when force was continuously increased from zero, the filling factor curve showed several ups and downs due to the abrupt appearance of a few large-amplitude MUP spikes (see Fig. 2, left). This complex profile of the filling factor–force curve cannot be accurately tracked when force was increased in a step-wise fashion (see Fig. 2, right). In other words, when constant-force contractions of increasing intensity are produced, one can only sample the filling factor curve at specific discrete values of force, thereby obtaining a limited restricted view of the changes in the filling factor curve.

### Factors explaining the high variability in the sEMG PDF at contraction forces below 10% MVC

The high inter-individual differences in the sEMG PDF shape observed for contraction forces below 10% are essentially due to the fact that the sEMG activity at these force levels can range from a “pulsatile” pattern (a few large-amplitude MUP spikes, clearly standing out from the background activity) to a “continuous” pattern (many small-amplitude MUPs with a great overlap between them), as shown in Fig. 4. How close the sEMG activity is to a “pure pulsatile” pattern or to a “pure continuous” pattern will determine whether the PDF approximates more to the semi-degenerate distribution (filling factor values ~ 0.25) or to the Gaussian distribution (filling factor values ~ 0.63), respectively [17].

The high diversity of sEMG activity at low contraction forces is due to several factors influencing the generation of the sEMG signal, as explained below:

1. Differences in the volume conductor characteristics, and especially in the subcutaneous layer thickness, which vary from one subject to another [19]. It has been found that, in muscles with a “thin” subcutaneous layer (< 5 mm), the motor units located superficially in the muscle would generate MUPs with much greater amplitude than those located at deeper regions, thus favoring the occurrence of a “pulsatile” sEMG activity [20, 21]. In contrast, in muscles with “thick” subcutaneous layers (> 10 mm), there would be only moderate differences in MUP amplitude between the motor units located at superficial and deep regions of the muscle: this scenario favors the generation of a “continuous” sEMG activity [20, 21].
2. Differences in the positioning of the EMG electrodes [24]. The active motor units contributing to the sEMG signal are determined by the sampling volume (i.e., the location) of the surface EMG electrodes.
3. Differences in the spatial distribution of motor units throughout the muscle cross-section [22, 23]. At present, there is no consensus on whether motor units are regionalized within the muscle according to their sizes or not, and if so, how this “regionalization” occurs. Indeed, in the *vastus lateralis* there are studies suggesting that smaller motor units with lower recruitment threshold primarily locate in deeper muscle regions, and larger units lie in more superficial regions [22], whereas the opposite spatial distribution has been reported in the *biceps brachii* [23].
4. Differences in the distribution of motor unit sizes between subjects. It has been recently demonstrated that muscle fiber diameters (and thus motor unit sizes) are not clustered into distinct groups (corresponding to motor unit types I and II), but rather, fiber diameter increases linearly with force, thus indicating a continuum of muscle fiber sizes [24]. A high dispersion in fiber diameter is observed around this linear regression line (see Fig. 4 of the cited paper of [24]), which means that a large motor unit can be recruited before all small motor units have been recruited.
5. Differences in the noise level during the recordings. An increase in noise amplitude (above 7–8 μV peak-to-peak) increases the likelihood of recording a “continuous” sEMG activity at low force levels [18].

Based on the above, the necessary conditions for the generation of a “pulsatile” sEMG activity (and thus a semi-degenerate PDF) at low contraction forces are the following: (1) a muscle with a “thin” subcutaneous layer; (2) in which large motor units are predominantly located in superficial regions; (3) where one or a few large motor units are recruited at a low recruitment threshold; and (4) noise level is below 7–8 μV. These 4 conditions are most likely fulfilled in many *vastus lateralis* of male subjects, as witnessed by the low filling factor values (0.3–0.4) found in this muscle (see Fig. 3). If one or more of these conditions are not fulfilled, or are partially fulfilled, then the sEMG activity will fall somewhere between the “pulsatile” and the “continuous” pattern, or what is the same, the sEMG PDF will fall somewhere between the semi-degenerate and Gaussian distribution, thus explaining the high variability in the PDF shape.

There is yet another factor that explains why the behavior of sEMG PDF with increasing contraction force varied greatly among subjects for forces below ~ 10% MVC. In our previous research we demonstrated that, at low contraction forces, the abrupt onset of a few large-amplitude MUP spikes (clearly standing out from the previous sEMG activity) makes the sEMG PDF abruptly shift from Gaussian towards semi-degenerate, thus inducing an abrupt drop in the filling factor [18]. In the present study, the great diversity of behaviors of the filling factor with contraction force up to ~ 10% MVC was largely due to the differences in force level at which this new “outstanding MUP train” abruptly appeared as force was increased.

### Factors explaining the low variability in the sEMG PDF for contraction forces above 10% MVC

A question remains to be answered: Why the sEMG activity at contraction forces above 10% MVC becomes largely “continuous” for all subjects? Or in other words, why the sEMG PDF approaches Gaussianity at forces greater than 10% MVC (as shown in Fig. 4, right)? Clearly, the explanation cannot be due to the subcutaneous layer thickness or to a specific spatial distribution of the motor units. The most likely reason is that, beyond a certain force threshold (~ 10% MVC), a higher number of motor units with different sizes have already been recruited throughout the entire muscle cross-section, both in the superficial and deep portions. With so many active motor units contributing to the electrode, it is less likely that a newly recruited motor unit potential stands out in amplitude from the previous EMG activity, i.e., it is less likely to generate a pulsatile sEMG activity.

### Why the high variability in the sEMG PDF at low contraction forces has passed unnoticed in previous studies

To our knowledge, this is the first study to report a high variability in the sEMG PDF shape at contraction forces below 10% MVC. Two main factors have prevented such observation in the past. First and most important, previous studies analyzed the PDF–force relation for contraction forces above 10% MVC, when the sEMG signal was already largely filled up, and thus when the PDF shape was rather similar (between Laplacian and Gaussian) among subjects [2, 11, 16]. As a second factor, in previous research, the PDF shape was examined for a limited number of constant-force contractions of different intensities, which hampered the full appreciation of the variability in the PDF shape [6, 11].

### Group analysis of the filling factor

Our group data indicate that, for contraction forces above 10% MVC, the shape of the sEMG PDF fell between the Laplacian (FF = 0.5) and Gaussian (FF = 0.63) distributions, a result in agreement with the majority of previous studies on the field [6, 9–11, 16]. Moreover, our results support the general notion that an increase in contraction force (above 10% MVC) shifts the sEMG PDF towards a Gaussian distribution, in line with the prevailing body of evidence [11, 12, 16]. Our group data also show for the first time that, for contraction forces below 10% MVC, the sEMG PDF can range between the semi-degenerate (FF = 0.25) and Gaussian (FF = 0.63) distributions, with a high variability among subjects, as explained above.

Importantly, the conclusion drawn by some authors that “the PDF of the sEMG signal recorded at low forces (around 10% MVC) is closer to a Laplacian than to a Gaussian” must be revisited in view of the present findings [11]. First, at 10% MVC, the filling factor had an average value of 0.55, which is approximately half-way between the Laplacian (0.5) and Gaussian (0.63) distributions. Second, we found that at 10% MVC there is considerable variability in the filling factor, which means that a PDF close to a Gaussian may found in some subjects for these low forces. In addition, the sEMG PDF at low contraction forces may differ depending on the muscle considered. Therefore, whether the “PDF is closer to a Laplacian or to a Gaussian” will ultimately depend on the subject and muscle tested. Hence, the above statement of Nazarpour et al. (2014) is a misleading oversimplification that trivializes the impact of the factors influencing the sEMG PDF shape [6].

Finally, one must be extremely careful when interpreting the average curve of the filling factor vs contraction force extracted from pooled data, as depicted in Fig. 6. This average curve could lead to the misleading conclusion that, for a given subject, the filling factor increases monotonically from ~ 0.35 to 0.63 as contraction force is increased from zero (as depicted in Fig. 6), i.e., that the sEMG PDF evolves monotonically from a semi-degenerate to a Gaussian distribution. However, analysis of individual data revealed that this is not so (see the different patterns illustrated in Fig. 3).

### Implications

The present results, and more specifically the high variability in the sEMG PDF shape encountered for contraction forces below 10% MVC, may have direct implications in several disciplines. For example, in prosthesis control, the high variability in the PDF shape at the beginning of the contraction will increase the complexity in the determination of the onset of myoelectric activity [25]. Indeed, in those individuals with an initial PDF shape close to a semi-degenerate (i.e., sEMG activity with a high signal-to-noise ratio), discriminating the sEMG signal from background noise would be much easier than in those individuals with initial PDF shape close to a Gaussian (i.e., sEMG activity with a low signal-to-noise ratio). Also, since the profile variation of the sEMG PDF shape with contraction force is subject-specific, algorithms for prosthesis control may have to be tailored for each subject.

The present findings would also have significance for biofeedback experiments where, in some cases, a certain PDF distribution has to be adopted to predict the envelope of the EMG signal [26]. Because the PDF shape can oscillate back and forth between the semi-degenerate and the Gaussian distribution for forces below 10% MVC, it would be difficult/inaccurate to choose a specific PDF to track the sEMG amplitude at such forces.

It must be stressed that, given the high variability of the PDF shape at low contraction forces reported here, any recommendation to adopt/choose a specific PDF shape to model the sEMG activity at such forces should be disregarded. In this respect, the interpretation offered by some authors that “the PDF of the sEMG signal resembles more to Laplacian than to a Gaussian distribution” should be treated with caution [6, 26]. For the same reason, any method that implicitly assumes that the sEMG PDF shape evolves monotonically towards Gaussianity as contraction force increases, such as the Motor Unit Number Index (MUNIX) method [27], would yield inaccurate outcomes.

Finally, it is important to emphasize that the differences in the PDF–force relation at low contraction forces among subjects are mainly due to two factors: (1) the thickness of the subcutaneous layer and (2) the position of the EMG electrodes. With regard to the first factor, muscles with thin subcutaneous layers (< 5 mm) would more likely present a pulsatile sEMG activity at low contraction forces, irrespective of the EMG electrode position. This pulsatile sEMG pattern has been found for the *vastus lateralis* and *vastus medialis* of male individuals (thin subcutaneous layer), whereas in the female counterparts (thicker subcutaneous layer), a continuous sEMG pattern was observed [20]. With regard to the second factor, the location of the EMG electrodes determines the detection volume-area in the muscle, and thus the active motor units contributing to the sEMG signal. This is a critical factor since, if one or a few large motor units located close to the recording electrodes are recruited at a low recruitment threshold, this could dramatically change the pattern of the EMG activity to pulsatile. Therefore, changing the placement of the bipolar electrodes in the same muscle would affect the PDF–force relation, but mainly for low contraction forces.

## Conclusions

Several conclusions emerged from the present study. The study demonstrated that the dependence of the sEMG PDF shape on contraction force can only be reliably assessed by gradually increasing force from zero, and not by performing a few constant-force contractions. We found that, in 84% of the subjects, the sEMG PDF shape did not evolve monotonically towards Gaussianity as force increased from 0 to ~ 10% MVC, but rather it oscillated back and forth between the semi-degenerate and the Gaussian distribution within such force range. The study also showed that the behavior of the sEMG PDF shape with increasing contraction force varied greatly among subjects for forces between 0 and ~ 10% MVC. For this reason, the widespread assumption that “the PDF of the sEMG signal recorded at low forces is closer to a Laplacian than to a Gaussian” is an oversimplification that should be treated with caution.

## Methods

### Participants

G-Power software was used to calculate sample size. The software indicated that, for a statistical power (1-β) of 0.90, a sample size of 15 participants was needed to detect differences in the filling factor across different contraction forces. Twenty-six male participants aged between 20 and 27 years (mean ± SD: 22 ± 2 years) were engaged in the study. Their average height and body mass were, respectively, 183 ± 5 cm and 74 ± 4 kg. The reason why females were not included as participants was that, due to the thick subcutaneous layer thickness of their *vastus lateralis*, the sEMG activity at low contraction forces was largely “continuous” (i.e., many MUPs greatly overlap with each other) for these participants, which impeded the analysis of the relationship between contraction force and filling factor. Written informed consent was obtained from all study participants before the experiments. None of the subjects reported any neuromuscular or current/recent musculoskeletal injuries. The experiments were performed according to the Declaration of Helsinki and approved by the Ethics Committee Board of the Public University of Navarra, Spain (PI-010/21).

### Experimental setup and force recording

Experiments involved the quadriceps muscle and consisted on gradually increasing the isometric knee extension force. During the experiments, participants were comfortably seated on a custom-built chair with a trunk–thigh angle of 100° and a knee angle of 90°. Extraneous movements of the upper body were limited by two crossover shoulder harnesses and a belt across the lower abdomen. Quadriceps force was recorded during the gradually increasing isometric contractions using a strain gauge (STS, SWJ, China, sensitivity 2 mV/V and 0.0017 V/N, linear range: 0–2452 *N*) that was attached to the chair and securely strapped to the ankle with a custom-made mold. The force signal (from the isometric knee extension) was digitized at 1000 Hz using an analog/digital converter (MP150; BIOPAC, Goleta, CA, USA).

### Localization of the innervation zone and the muscle fibers’ direction

Determination of the innervation zone position and muscle fibers’ direction in the *vastus lateralis* was made by means of a dry linear array of 16 electrodes (5 mm inter-electrode distance). The linear array was connected to a multichannel amplifier (OT Bioelettronica, Torino; bandwidth 10–500 Hz). The sEMG signals were registered as the participant performed gentle isometric contractions under single-differential (bipolar) configuration. The location of the innervation zone was that corresponding to the channel of the array showing minimum amplitude or phase reversal [28]. The direction of the muscle fibers corresponded to the orientation of the array that yielded optimal propagation of action potentials between the innervation zone and tendon regions [29].

### Electromyographic recordings

Surface EMG potentials were recorded from the *vastus lateralis* using self-adhesive electrodes (Ag/AgCl, Kendall Meditrace 100) with a circular shape (recording diameter, 10 mm). Recordings were made using a pair of electrodes arranged in bipolar configuration, with an inter-electrode distance of 20 mm. Specifically, the proximal electrode of the pair was placed over the innervation zone, and the other electrode was placed distally along the direction of the muscle fibers, as previously done [30]. The “ground” electrode was located over the patellar tendon. Surface EMG signals were amplified (gain, 500 V/V; bandwidth, 10 to 5000 Hz) and digitized (sampling frequency of 5000 Hz) using an analog/digital converter (MP150; BIOPAC, Goleta, CA). Subsequently, a second-order Butterworth 10—1000 Hz was applied to the signal. The maximum acceptable noise amplitude (peak-to-peak) was 5–6 μV as previously established [18].

### Experimental protocol

The participants were all familiarized with the experimental procedures, since they had been involved in previous experiments. All participants were asked not to engage in heavy physical exercise 24 h before the testing appointment.

On the experiment day, participants completed several warm-up contractions. Subsequently, they performed 3 brief (4 s) maximum voluntary contractions (MVCs), with a 3 min resting period in between. The average peak force from these three MVCs were averaged to determine each subject’s MVC force: this average maximal force was used to calculate the force trajectory during the force protocols (see below).

The experiments consisted of two force protocols performed in random order and separated by at least 10 min (see Fig. 2). In one force protocol, subjects were asked to perform one ramp contraction of 60 s, in which force was increased gradually and linearly from 0 up to 20% of MVC force during the first 30 s, and then from 20 up to 80% MVC force during the last 30 s of the contraction (between 30 and 60 s). The desired force trajectory was displayed on a computer screen along with the output of the force transducer. The rate of force increase during the first half of the contraction (up to 20% MVC) was intentionally slow because the filling process of the sEMG signal is known to be very fast, with the sEMG being nearly filled at ~ 10% MVC [18]. In the other force protocol, subjects were asked to perform step-wise contractions at the following force levels: 0.5, 2.5, 5, 10, 20, 40, 60, and 80% of their MVC force. The order of contraction intensity was randomized (see Fig. 2). The duration of each contraction was 5 s, and a rest period of 2 min was allowed between consecutive contractions. Participants were presented a target force level in the screen for each contraction force.

### Definition of the EMG filling factor

The filling factor is an index determined by the shape of the sEMG PDF distribution, which informs of the degree to which an EMG signal has been filled [17]. Specifically, this index is calculated from the first two non-central of the rectified sEMG signal, which are defined as follows:

$${m}_{1}=\frac{1}{N}\sum_{\text{n}=0}^{N-1}\left|\text{x}\left[\text{n}\right]\right|,$$

$${m}_{2}=\frac{1}{N}\sum_{\text{n}=0}^{N-1}{\left|\text{x}\left[\text{n}\right]\right|}^{2},$$

where x[n] is the sampled sEMG signal and *N* is the number of samples in each recording. Note that *m*_1_ represents the rectified sEMG mean, whereas *m*_2_ is the square of the sEMG root mean square (RMS). Then, the EMG filling factor (FF) is calculated as the ratio between *m*_1_^2^ and *m*_2_ as follows:

$$\text{FF}=\frac{{{m}_{1}}^{2}}{{m}_{2}}.$$

### Analysis of the changes in the sEMG PDF shape with contraction force

To examine how the shape of the sEMG PDF changes with increasing contraction force, we analyzed the changes in the filling factor as a function of force. In the first force protocol (ramp contraction), the filling factor was calculated over successive non-overlapping windows of the sEMG signal during the 60-s contraction. Window duration was 0.8 s, similar to our previous report [18]. In the second force protocol (step-wise contractions), the filling factor was calculated over three consecutive 0.8-s windows centered around the middle of each contraction (during which the force was approximately constant), and the average filling factor was obtained. Good-to-excellent reliability (intra-class correlation coefficient > 0.7) was found in the filling factor calculated across the three consecutive 0.8-s windows.

Insight into the variability in the relation between sEMG PDF and contraction force among subjects (i.e., inter-individual differences) was obtained by using the Dynamic Time Warping (DTW) algorithm, an index that measures the similarity between two temporal sequences (see statistics section for details). DTW was used instead of a standard mean squared error to take into account the possible shifts in the sequences. In addition, we sought whether the variation of the filling factor with force fell into different groups or patterns.

An objective of the present investigation was to determine whether there were marked fluctuations of the subjects’ sEMG PDF shape between the semi-degenerate and Gaussian distributions for contraction forces below 10% MVC. To identify and objectivize the presence of such marked fluctuations, we set the following criterion: a fluctuation in the filling factor occurs when this index falls from a value above 0.5 to a value below 0.4 in less than four consecutive 0.8 s windows.

### Statistics

Kolmogorov–Smirnov tests confirmed that the filling factors assessed at different force levels were normally distributed (*p* < 0.05). To assess the degree of inter-subject variability in the relationship between filling factor and force among subjects we used the dynamic time warping (DTW) algorithm, a technique for measuring similarity between two temporal sequences. Since there is no gold standard filling factor–force curve to compare with, we first calculated the average filling factor–force curve from all subjects, and then, computed the DTW distance between each subject’s curve and the average curve. The calculation of the DTW distance was made separately for the force intervals (0–10), (10–20), (20–30), (30–40), (40–50), (50–60), (60–70), and (70–80)% MVC. Then, the average DTW distance for each individual force interval was calculated from all subjects. The changes in the DTW distance across the difference force intervals [(0–10), (10–20), …, and (70–80)% MVC] were investigated with a one-way repeated-measures ANOVA. Effect size of the mean differences was determined using Cohen’s *d*. To examine the effects of contraction force on the sEMG PDF, a two-way repeated-measures ANOVA [relative contraction level (0.5, 2.5, 5, 10, 20, 40, 60, and 80% of MVC force) × force protocol (ramp vs step-wise)] was performed on the filling factor. When main effects or interactions were significant, Student–Newman–Keuls post hoc tests were conducted. Statistical significance was set at *p* < 0.05. Data were presented as mean ± SD in the text, tables and figures.

## Data Availability

No datasets were generated or analysed during the current study.

## Abbreviations

EMG: Electromyography
MVC: Maximal voluntary contraction
PDF: Robability density function
